# Fate Bifurcation of Cellular Senescence: Dynamic Regulation from Tumor Suppression to Recurrence Risk

**DOI:** 10.3390/cells15121123

**Published:** 2026-06-22

**Authors:** Xiuhong Chen, Huilong Liu, Qipeng Shu, Yuntao Tang, Jia Zhang, Weizhe Yu, Shangze Li

**Affiliations:** 1 School of Medicine, Chongqing University, Chongqing 400030, China; 2 College of Medicine, Xizang University, Lhasa 850000, China

**Keywords:** cellular senescence, senescence-associated secretory phenotype, tumor recurrence, cancer therapy

## Abstract

Cellular senescence is a state of stable cell cycle arrest triggered by various internal and external stressors. It represents an important tumor-suppressive mechanism that effectively prevents the proliferation of damaged cells. During tumor initiation and progression, cellular senescence plays a dual and paradoxical role. On one hand, it induces cell cycle arrest to inhibit the development of tumors in potentially malignant cells. On the other hand, it can promote tumor progression through the senescence-associated secretory phenotype (SASP), which enhances inflammation and extracellular matrix remodeling. This review outlines the definition and key characteristics of cellular senescence and analyzes different senescence-inducing stimuli along with their underlying molecular mechanisms. It further discusses the molecular basis for the maintenance of stable senescence, mechanisms to escape growth arrest, and how these cells contribute to tumor recurrence through dedifferentiation and acquisition of stemness properties. Additionally, the dual regulatory role of SASP in tumor progression is examined. In terms of cancer therapy, with a deeper understanding of the mechanisms of senescent cells, treatment strategies are gradually shifting from single senescence-inducing approaches to more comprehensive combinatorial strategies. Meanwhile, the integration of single-cell omics technologies with artificial intelligence and machine learning offers new prospects for personalized therapy.

## 1. Introduction

Cellular senescence is a state of stable cell cycle arrest to inhibit continued cell proliferation when cells are subjected to endogenous or exogenous stress or damage, and it is an important tumor-suppressive barrier in the organism. Senescent cells exhibit distinct phenotypic alterations, including a flattened morphology, nuclear enlargement, epigenetic remodeling, and metabolic changes [1,2]. They also display cell non-autonomous activities, particularly the secretion of a variety of cytokines, chemokines, growth factors, immune modulators, proteases, and matrix metalloproteinases (MMPs), a phenotype collectively referred to as the senescence-associated secretory phenotype (SASP) [3].The concept of cellular senescence can be traced back to the seminal discovery by Hayflick and Moorhead in 1961, which demonstrated that normal human fibroblasts undergo a finite number of divisions in vitro before entering an irreversible state of growth arrest, and it is known as replicative senescence [4]. In recent years, studies have revealed that cellular senescence can be induced by a wide range of endogenous and exogenous stressors, including replicative stress, telomere dysfunction, metabolic imbalance, oncogenic stimuli and others, and all of these factors can trigger distinct types of senescence [5].

Cellular senescence presents a dual and contradictory role. In one aspect, a stable senescent state enforces cell cycle arrest through canonical pathways such as p53/p21 and p16INK4a/Rb. In addition, the senescence-associated secretory phenotype (SASP) mediates the recruitment of immune cells and facilitates senescence surveillance, promoting the recognition and clearance of potentially tumorigenic cells and exerting a critical tumor-suppressive function [6,7,8]. In another aspect, senescence is not invariably a stable and irreversible endpoint. More and more evidences indicate that under specific genetic, epigenetic, or therapeutic pressures, a subset of senescent cells can escape from senescence and re-enter the cell cycle [9]. This process is often accompanied by the attenuation or inactivation of key tumor suppressor pathways, remodeling of chromatin architecture, and alterations in epigenetic regulation, ultimately leading to stem cell-like properties. Cells that undergo the senescence-to-proliferation transition often show increased invasiveness and resistance to therapy. These properties may promote tumor progression and recurrence [10,11,12].

In the context of cancer therapy, therapy-induced senescence displays dual effects. Various anticancer treatment modalities, such as chemotherapy, radiotherapy, and targeted therapies, can drive tumor cells into a senescence-like growth arrest, to limit tumor progression [13]. However, if these therapy-induced senescent cells are not effectively cleared, they may persist long-term and form residual lesions. Under the combined influence of chronic inflammation, immunosuppression, and the remodeling of the tumor microenvironment, these cells may regain proliferative capacity and serve as an important source of tumor recurrence and metastasis. The continuous secretion of SASP may lead to malignant transformation in neighboring cells. This effect can further increase tumor heterogeneity [14,15,16]. Considering the points discussed above, cellular senescence represents a key bifurcation point. Cells at this stage can follow a tumor-suppressive trajectory, marked by stable growth arrest and immune-mediated clearance. Alternatively, under certain conditions, cells may evade senescence and transition toward tumor-promoting processes.

In this review, we examine the molecular basis underlying stable cellular senescence as well as the mechanisms to evade growth arrest. We further explore the dual regulatory role of SASP in tumor progression. In addition, we analyze both intrinsic and extrinsic factors that influence tumor cell fate decisions. We also examine therapeutic strategies designed to either induce senescence or selectively eliminate senescent cells within tumors. Finally, we discuss emerging technologies such as single-cell omics, artificial intelligence, and machine learning, and these technologies offer new opportunities for developing personalized cancer therapies.

## 2. Types of Cellular Senescence

Cellular senescence is a stable state of cell cycle arrest that can be triggered by a variety of intrinsic and extrinsic stimuli. Depending on the initiating factor, senescence encompasses multiple types, including telomere damage-induced senescence, oncogene-induced senescence (OIS), therapy-induced senescence (TIS), and senescence resulting from mitochondrial dysfunction and so. These stimuli can activate the DNA damage response (DDR) to different extents. Activation of DDR subsequently engages downstream pathways, including p53–p21 and p16–Rb, which influence cell cycle arrest [17,18,19,20].

### 2.1. Replicative Senescence

Replicative senescence is primarily driven by the progressive shortening of telomeres. Conventional DNA replication cannot fully copy the terminal sequences of linear chromosomes. As a result, telomeres gradually shorten with each round of cell division. When telomere function becomes compromised, it is recognized as DNA damage, leading to the persistent activation of the canonical DNA damage response (DDR), which is predominantly mediated by ATM [21,22]. In this context, telomere dysfunction induces senescence mainly through the p53–p21 pathway, which inhibits CDK2 activity and mediates early cell cycle arrest. With sustained stress, p16^INK4a^ is progressively upregulated, leading to the hypophosphorylation of RB and subsequent suppression of E2F-dependent transcriptional programs. This process establishes and reinforces a stable cell cycle arrest. The p16 pathway is considered to provide a second proliferative barrier in response to telomere dysfunction [23,24,25,26,27]. Replicative senescence is more likely to result in a stable state of cell cycle arrest and contributes to tissue and organismal aging through the accumulation of senescent cells.

### 2.2. Oncogene-Induced Senescence

Oncogene-induced senescence (OIS) represents a critical cellular stress response to aberrant oncogenic signaling, such as sustained activation of RAS or BRAF, and it is considered an important intrinsic barrier during the early stages of tumorigenesis. Oncogene-driven hyperproliferation can induce replication stress and associated cellular stress, leading to DNA damage and the activation of the DNA damage response (DDR) [28]. The coordinated action of the p53–p21 and p16^INK4a^–RB signaling axes establishes a stable cell cycle arrest [29,30]. In addition, OIS is frequently associated with pronounced chromatin remodeling. This includes the formation of senescence-associated heterochromatin foci (SAHFs) in a subset of senescent cells and changes in cellular metabolism [31,32,33]. OIS is generally regarded as a key early tumor-suppressive barrier that prevents malignant transformation by restricting oncogene-driven aberrant proliferation [34]. However, its tumor-suppressive function is context-dependent. When the p53 or RB pathways are compromised, or when cells acquire the ability to escape senescence, the OIS barrier can be bypassed, consequently promoting tumor progression [35,36].

### 2.3. Therapy-Induced Senescence

Therapy-induced senescence (TIS) is a form of cellular senescence triggered by anticancer treatments such as radiotherapy, chemotherapy, and targeted therapies. It is typically initiated by sublethal DNA damage, leading to prolonged or even stable cell cycle arrest, with features resembling those of replicative senescence and oncogene-induced senescence [13,37]. In the short term, TIS cells can function as a tumor-suppressive barrier by inhibiting tumor cell proliferation, and in some cases, enhancing treatment sensitivity, which contributes to improved therapeutic efficacy. Accordingly, certain therapeutic strategies aim to deliberately induce senescence, for instance, by activating key pathways such as p53–p21 and p16–RB or by inhibiting telomerase activity to reinforce the senescence response [12,38,39]. However, an increasing body of evidence indicates that TIS cells accumulating over time within the tumor microenvironment can exert context-dependent dual biological effects. With the secretion of SASP, these cells remodel the tumor microenvironment, promoting inflammatory responses, immune evasion, and tumor cell invasiveness, which may ultimately contribute to therapeutic resistance, tumor recurrence, and metastasis [15,40].

## 3. Molecular Mechanisms Underlying Stable Senescence and Its Tumor-Suppressive Functions

**Figure 1:**
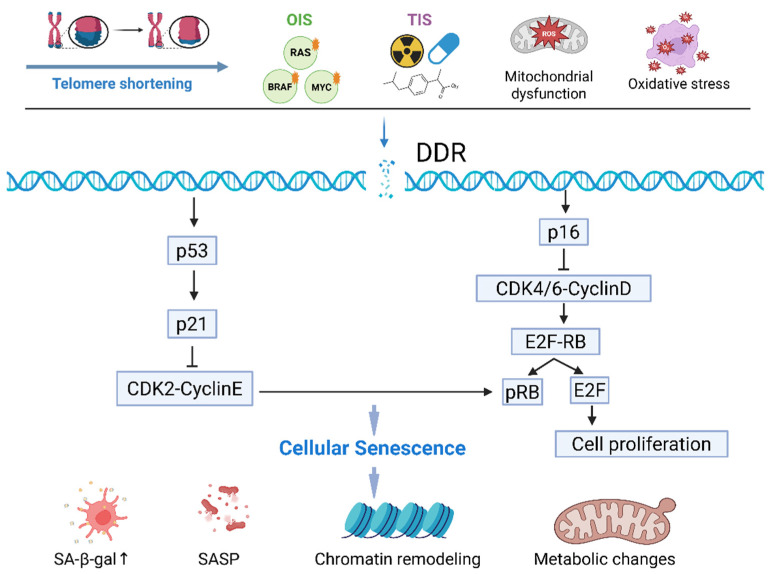
Molecular pathways controlling growth arrest during senescence. Cellular senescence pathways are triggered by various stressors, such as telomere shortening, oncogene activation, therapeutic stress, and other damage signals. Persistent DNA damage response (DDR) activates p53, leading to the upregulation of p21. Persistent DNA damage induces the expression of p16INK4a. These CDK inhibitors suppress E2F activity, leading to the cell cycle arrest. Senescent cells display several phenotypes, including chromatin remodeling, secretion of SASP factors, and metabolic alterations.

One of the primary functions of cellular senescence is to limit the continued proliferation of damaged cells through stable cell cycle arrest. This process is predominantly regulated by two key pathways: the p53–p21 and p16–Rb axes [41,42]. Various factors that induce cellular senescence often cause DNA damage to varying degrees. ATM and ATR are key kinases in the DNA damage response (DDR) pathway, with ATM primarily recognizing DNA double-strand breaks (DSBs) and ATR mainly responding to single-stranded DNA damages. Activated ATM/ATR phosphorylate downstream kinases CHEK1 and CHEK2 which in turn activate the tumor suppressor p53 and induce the expression of the cyclin-dependent kinase inhibitor p21. p21 suppresses the activity of CDK complexes, such as CDK2, thus restricting the G1/S transition [8,43,44]. Simultaneously, under certain conditions, cell cycle arrest or stress signaling can upregulate p16, which inhibits CDK4/6 and maintains Rb in a hypophosphorylated state. This enhances the Rb-mediated repression of E2F transcription factors and further reinforces cell cycle arrest [27,45]. Notably, p21 is activated during the early phase of senescence, whereas p16 becomes prominent at later stages, likely to sustain the senescent phenotype. In addition, persistent DDR signaling, including the activation of γ-H2AX and CHEK1/2, further reinforces the senescent state [46]. Figure 1 illustrates the molecular mechanism of cellular senescence.

Stable senescence not only prevents the proliferation of potentially oncogenic cells through sustained cell cycle arrest but also relies on immune surveillance mechanisms to maintain its tumor-suppressive function. It is also accompanied by a complex senescence-associated secretory phenotype (SASP), comprising factors such as IL-6, IL-8, CCL2, and matrix metalloproteinases (MMPs) [47]. The production of SASP depends on the activation of NF-κB and cGAS–STING signaling pathways. These secreted factors establish a pro-inflammatory microenvironment that recruits immune cells—including natural killer (NK) cells, macrophages, and T cells—thereby promoting the immune recognition and clearance of senescent cells [48,49].

Immune surveillance not only eliminates damaged cells but also plays a crucial inhibitory role in early tumorigenesis. Through chemotaxis and the activation of immune cells, SASP facilitates the recognition and clearance of senescent cells by the immune system, thus preventing potential malignant transformation. However, when immune surveillance is impaired or the clearance of senescent cells is hindered, prolonged accumulation of SASP can lead to chronic inflammation and the disruption of tissue homeostasis, which may instead promote tumorigenesis. Therefore, maintaining a dynamic balance between senescence induction and clearance—through the regulation of key molecular pathways and the promotion of immune-mediated elimination—is essential for preserving its tumor-suppressive effects [50].

## 4. Reversibility of Senescence and Senescence Escape

Long-term cellular senescence has been considered a stable and irreversible state of cell cycle arrest, acting as an important barrier against tumorigenesis in organisms. However, accumulating evidence indicates that cellular senescence exhibits a certain degree of plasticity. Under specific conditions, cells may undergo senescence escape, allowing a subset of cells that were previously in a senescent state to re-enter the cell cycle and regain proliferative capacity [12,51]. Such renewed proliferation may arise either from the reversal of a truly senescent state or from the selective expansion of cell subpopulations that are resistant to senescence induction. Among these, therapy-induced senescence (TIS) provides important evidence supporting this phenomenon [40]. Various chemotherapeutic agents, radiotherapy, and certain targeted therapies can induce tumor cells to enter a senescent state. Subsequent studies, however, have shown that a fraction of residual cells can resume proliferation after the alleviation of stress. Notably, these escaped cells often display enhanced clonogenic capacity, genomic instability, increased invasiveness, and stronger drug resistance, and may represent a cancer stem-like cell subpopulation. At the same time, given the heterogeneity of tumor cell populations, not all cells undergo therapy-induced senescence (TIS). Certain subpopulations may evade the senescent state and retain their proliferative potential. Consequently, cellular senescence may constitute a transient intermediate state during tumor progression and recurrence, rather than an irreversible terminal fate [10,52].

At the molecular level, senescence escape is commonly associated with the functional attenuation of key tumor suppressor pathways. Cancer-associated mutations frequently occur in critical tumor suppressor proteins involved in the regulation of senescence, such as p53, p16INK4a, and Rb; these alterations are considered fundamental to the ability of cells to bypass the senescence barrier. Mutations in p53 or the inhibition of its signaling pathway can impair the transmission of DNA damage responses, thereby weakening the p21-mediated cell cycle arrest pathway. Meanwhile, the downregulation of p16INK4a expression or inactivation of the Rb pathway can restore CDK4/6 activity, promoting the transition from G1 to S phase [53,54]. In addition, epigenetic remodeling plays a critical role in this process. Characteristic heterochromatin structures in senescent cells, such as senescence-associated heterochromatin foci (SAHF), may undergo structural alterations under specific conditions. These changes are accompanied by dynamic modifications in histone marks, including H3K9me3 and H3K27me3, as well as alterations in DNA methylation patterns. Together, these epigenetic changes can relieve the repression of proliferation-associated genes and promote their re-expression [55,56,57].

## 5. Senescence Escape-Driven Plasticity and Stemness Acquisition

The phenotypic transformation observed in cells following senescence escape can be regarded as a complex regulatory network involving processes such as dedifferentiation, the acquisition of stem-like properties, and epithelial–mesenchymal transition (EMT). This process is characterized by pronounced cellular plasticity and plays a critical role in tumor progression, metastasis, and therapeutic resistance.

### 5.1. Stemness and Plasticity Following Senescence Escape

Cancer stem cells (CSCs) represent a subpopulation of tumor cells with a low degree of differentiation, possessing strong self-renewal capacity and tumorigenic potential and they are also referred to as tumor-initiating cells or tumor-propagating cells [58]. Within the tumor microenvironment, cellular senescence is increasingly recognized as a highly plastic and dynamic process, in which a subset of senescent cells can acquire regenerative potential through the reprogramming of stemness-associated transcription factors. Specifically, the activation of key stemness regulators such as WNT/LEF1, OCT4, and NANOG can drive cells toward a less differentiated state and promote their re-entry into the cell cycle, therefore facilitating dedifferentiation and the acquisition of stem-like properties. This process enables cells that were previously terminally differentiated or senescent to acquire CSC-like characteristics, giving rise to a cancer stem cell-like phenotype [10,59]. Numerous studies have demonstrated that a variety of epigenetic modifications play central roles in CSC plasticity, including DNA methylation, chromatin remodeling, and histone modifications [60]. In addition, the senescence-associated secretory phenotype (SASP) enhances tumor cell stemness and proliferative capacity through the paracrine secretion of inflammatory factors such as IL-6, IL-8, and IL-1α, thereby promoting tumor progression [15].

### 5.2. Epithelial–Mesenchymal Transition

Epithelial–mesenchymal transition (EMT) is widely recognized as one of the principal mechanisms regulating cancer stem cell (CSC) plasticity. EMT is a multistep and dynamic process of cellular reprogramming, characterized by the progressive loss of epithelial features and the acquisition of mesenchymal phenotypes [61]. This process plays a pivotal role in tumor progression by altering cell–cell interactions and activating gene expression programs that promote the mesenchymal state, thus driving epithelial cell depolarization and enhancing migratory capacity. EMT is closely associated with multiple hallmarks of tumor biology, including intravasation, tumor initiation, cell migration, maintenance of CSC properties, metastasis, and resistance to chemotherapy or targeted therapies [62]. At the molecular level, EMT is coordinately regulated by multiple signaling pathways, among which the TGF-β and Wnt/β-catenin pathways serve as key drivers. Under the influence of these signals, EMT-associated transcription factors such as Snai1, Slug, Twist, and Zeb1 are induced or activated, thereby initiating EMT-associated gene expression programs and mediating dynamic phenotypic reprogramming [58,63,64]. These regulatory mechanisms are highly conserved across species, highlighting the fundamental biological significance of EMT in tumor cell plasticity and the acquisition of stemness.

## 6. Therapy-Induced Senescence and Mechanisms of Tumor Recurrence

### 6.1. Mechanisms of TIS-Driven Minimal Residual Disease and Tumor Recurrence

During anticancer treatment, therapy-induced senescence (TIS) is increasingly recognized as a cellular response with dual biological effects. Chemotherapy, radiotherapy, and targeted therapies induce the DNA damage response (DDR), activating the p53–p21 or p16–Rb pathways, thereby driving tumor cells into a stable state of cell cycle arrest and transiently suppressing tumor growth [65]. However, unlike apoptotic cells, senescent cells remain metabolically active and can persist in tissues for prolonged periods, thereby posing a latent risk for subsequent tumor progression [66]. In the post-treatment setting, TIS is closely associated with tumor dormancy and minimal residual disease (MRD). MRD is generally defined as the presence of residual cancer cells in patients following treatment that is undetectable by conventional imaging techniques, representing a clinically occult stage of disease progression [67]. MRD can be detected using methods such as droplet digital polymerase chain reaction (ddPCR), next-generation sequencing (NGS), and flow cytometry [68,69]. A subset of senescent tumor cells can acquire long-term survival capacity through metabolic reprogramming, enhanced anti-apoptotic signaling, and maintenance of cancer stem cell (CSC) properties. These cells may enter a dormant state within tumor tissues while retaining the ability to overcome cell cycle arrest, thereby constituting MRD—residual tumor cell populations that persist during clinical remission but remain difficult to detect, being considered a key driver of tumor recurrence [70]. Under sustained therapeutic pressure, MRD does not represent a static population of residual tumor cells, but rather consists of multiple phenotypically heterogeneous subpopulations. These subpopulations exhibit dynamic behavior and phenotypic plasticity, governed by the interplay between intrinsic molecular programs and extrinsic signals from the tumor microenvironment. Together, these factors form the basis of therapeutic resistance and tumor relapse [71].

### 6.2. Activation of the Tumor Microenvironment, Inflammation, and Immune Evasion in Tumor Recurrence

The reactivation of therapy-induced senescent (TIS) cells is regulated by multiple factors, particularly chronic inflammatory signaling, immune evasion mechanisms, and remodeling of the tumor microenvironment (TME). A senescence-associated TME is typically characterized by immunosuppression and chronic inflammation, a phenomenon largely driven by the senescence-associated secretory phenotype (SASP) [72]. These microenvironmental changes not only favor tumor cell survival and dissemination but may also promote the reactivation of a subset of residual senescent cells under specific conditions, enabling their re-entry into the cell cycle and enhancing tumor regenerative potential [14,73]. Specifically, in the context of heightened inflammatory responses, impaired immune surveillance, or TME remodeling, senescent cells can escape their quiescent state and regain proliferative capacity. This process is regulated by the reactivation of multiple key signaling pathways, including Wnt/β-catenin and Hippo–YAP/TAZ signaling [74,75,76]. Concurrently, disruption of the p53 or Rb pathways relieves cell cycle constraints, further facilitating tumor recurrence [53].

In addition, TIS cells secrete SASP factors such as IL-6, IL-8, IL-1α, and CCL2, which mediate the activation of inflammatory signaling pathways. Among these, IL-6 and IL-8 have been shown to activate the STAT3 signaling pathway, thereby sustaining an immunosuppressive microenvironment that promotes tumor growth [77,78]. Meanwhile, immune evasion plays a critical role in tumor recurrence. SASP contributes to immune escape by recruiting immunosuppressive cell populations, including myeloid-derived suppressor cells (MDSCs) and regulatory T cells (Tregs), thereby facilitating tumor progression [16]. Furthermore, TIS cells can upregulate immunosuppressive molecules to inhibit the immune clearance functions of T cells and macrophages, thereby promoting immune evasion [79]. Notably, Hwang et al., in both murine tumor models and human cancer cell studies, demonstrated that TIS tumor cells can evade immune surveillance and promote tumor recurrence by upregulating the immune checkpoint molecule PD-L1, which suppresses CD8^+^ T cell-mediated cytotoxicity [80].

## 7. Stromal Cells and the Dual Roles of SASP in the Tumor Microenvironment

Tumor initiation and progression are not only determined by intrinsic genetic programs but also heavily rely on the integration of external microenvironmental signals. Under certain conditions, stromal cells in the tumor microenvironment can form a senescent niche and participate in local niche remodeling through the senescence-associated secretory phenotype (SASP). Among these, the senescence-associated secretory phenotype (SASP), one of the most prominent features of senescent cells, is highly dynamic in its composition and varies according to cellular context and stress conditions. SASP exhibits a dual “double-edged sword” nature. On one hand, during the early stages of tumorigenesis, SASP can exert tumor-suppressive effects by recruiting immune cells to facilitate immune surveillance and by inhibiting the proliferation of malignant cells. On the other hand, persistent SASP signaling can lead to the establishment of a pro-inflammatory microenvironment, which promotes tumor progression through mechanisms such as chronic inflammation, immune suppression, and extracellular matrix remodeling. Therefore, SASP represents a critical regulatory node linking the senescence program to tumor cell fate determination [15,81].

### 7.1. Stromal Cells in the Tumor Microenvironment

Stromal cells within the tumor microenvironment (TME) constitute a critical component in mediating and regulating the effects of cellular senescence. In addition to oncogene-induced senescence (OIS) and therapy-induced senescence (TIS) occurring in tumor cells themselves, non-malignant stromal cells, such as fibroblasts, endothelial cells, mesenchymal cells, and immune-associated stromal populations, can also undergo senescence in response to aging, chronic inflammation, tissue injury, oxidative stress, or therapeutic interventions. These senescent stromal cells contribute to the remodeling of local tissue niches through the secretion of senescence-associated secretory phenotype (SASP) factors, thereby influencing tumor cell survival, immune evasion, recurrence, and metastasis. Among these stromal components, cancer-associated fibroblasts (CAFs), one of the predominant cellular populations within the tumor stroma, are widely recognized as key contributors to the establishment and maintenance of the tumor microenvironment [73,82].

Notably, senescent stromal cells can modulate the interactions between cancer cells and their surrounding microenvironment by altering collagen deposition, fiber organization, matrix stiffness, and the expression of adhesion molecules. Such changes are considered highly relevant to the formation of the pre-metastatic niche [83]. Early studies largely viewed metastatic niches as the consequence of adaptive interactions between disseminated tumor cells and the microenvironment of distant organs. However, accumulating evidence suggests that alterations in host tissue microenvironments may precede tumor cell dissemination and actively facilitate metastatic colonization [84]. Perié et al. demonstrated that senescent ovarian fibroblasts promote ovarian cancer cell dissemination through SASP-mediated signaling and fibronectin-associated extracellular matrix (ECM) remodeling, enhancing cancer cell migration and invasion. These findings highlight senescent stromal cells as important drivers of age-related changes within the tumor microenvironment [85]. In parallel, senescent endothelial cells have been shown to increase vascular permeability and upregulate the expression of adhesion molecules, thereby facilitating the extravasation of circulating tumor cells. Collectively, these observations indicate that senescence-associated alterations in the microenvironment not only influence the progression of primary tumors but may also contribute to the initiation and establishment of metastatic disease [86].

### 7.2. Antitumor Effects: Tumor-Suppressive Functions During Early Tumor Initiation

One of the antitumor functions of SASP lies in its role in maintaining cell cycle arrest. Factors such as IL-6 and CXCR2 ligands secreted by senescent cells can reinforce cell cycle arrest through p53-dependent pathways, thereby inhibiting the proliferation of potentially tumorigenic cells [15,87]. In early or transiently induced senescence contexts, SASP typically exhibits a pro-inflammatory profile and is associated with immune-activating functional phenotypes. Pro-inflammatory cytokines and chemokines secreted by SASP—including IL-6, IL-8, CCL2, and CXCL10—promote the recruitment and activation of immune cells. Previous studies have shown that immune cells such as NK cells, T cells, and macrophages participate in the immune surveillance and clearance of senescent cells, thus limiting their accumulation and suppressing potential tumorigenesis [50,88,89]. For example, Yin et al. (2022) demonstrated in human liver sinusoidal endothelial cells (LSECs) that SASP can induce NF-κB activation in endothelial cells and drive immune cell recruitment [90]. Endothelial cells further promote immune-mediated senescence surveillance and clearance by regulating the formation of STAT1^+^ CD4^+^ T cells and the ICOS–ICOSLG costimulatory axis.

In addition, cytosolic DNA can activate type I interferon and inflammatory signaling through the cGAS–STING pathway, therefore amplifying SASP-associated inflammatory responses and enhancing immune recognition. Meanwhile, membrane-bound IL-1α, as an upstream regulator of SASP, can sustain the stability of the inflammatory SASP through a positive feedback loop [91,92]. Furthermore, SASP can induce paracrine senescence in neighboring cells, thereby propagating the senescence response within local tissues and limiting the proliferation of potentially aberrant cells, ultimately forming a tissue-level antitumor barrier [93].

### 7.3. Pro-Tumorigenic Effects: Chronic SASP-Driven Inflammation in Tumor Progression

When senescent cells are not efficiently cleared, the persistent secretion of the senescence-associated secretory phenotype (SASP) leads to the establishment of a chronic inflammatory microenvironment, during which SASP gradually shifts from a tumor-suppressive to a tumor-promoting role. At this stage, the composition of SASP undergoes significant changes, characterized by the upregulation of factors such as TGF-β, IL-10, and various matrix metalloproteinases (MMPs), which confer immunosuppressive properties and are associated with immunosuppressive functional phenotypes [15,94]. Under conditions of chronic or persistent senescence, SASP typically exhibits a pro-inflammatory profile and participates in the immunoregulation of the tumor microenvironment through multiple signaling pathways. NF-κB, a key transcriptional regulator of SASP, drives the expression of inflammatory cytokines such as IL-1β, IL-6, and TNF-α, thereby contributing to the maintenance of chronic inflammation. In this context, cytokines such as IL-6 and IL-8 can further activate the JAK–STAT signaling pathway, amplifying the inflammatory SASP response [81,95].

In tumor settings, IL-6/JAK/STAT3 signaling has been reported to modulate tumor antigen expression and suppress antitumor immune responses, which may contribute to T cell exhaustion [77,96]. In addition, SASP factors such as IL-1β can promote the polarization of macrophages toward an M2-like phenotype, while secreted factors including CCL2 and TGF-β facilitate the recruitment of myeloid-derived suppressor cells (MDSCs) and the expansion of regulatory T cells (Tregs), respectively, collectively shaping an immunosuppressive microenvironment [97,98]. Furthermore, Eggert et al. (2016), using a hepatocellular carcinoma (HCC) model, demonstrated that senescent tumor-adjacent tissues promote tumor growth by inducing immature myeloid cell-mediated suppression of natural killer (NK) cells through the CCL2–CCR2 signaling axis [89].

**Figure 2:**
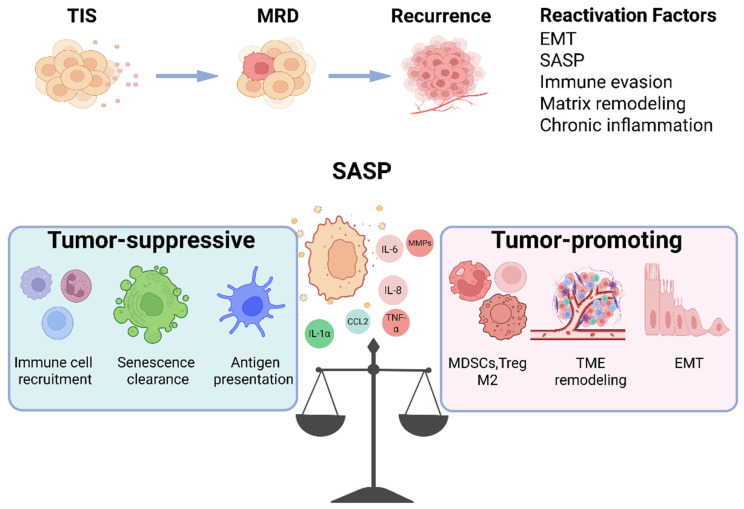
From senescence to recurrence: the role of SASP and microenvironment. Therapy-induced senescent cells can contribute to minimal residual disease (MRD) and tumor recurrence. SASP secretion mediates both tumor-suppressive effects, via immune surveillance, and tumor-promoting effects, through microenvironment remodeling, chronic inflammation and epithelial–mesenchymal transition (EMT).

Emerging evidence suggests that, in specific contexts, SASP induces PD-L1 upregulation in tumor cells, facilitating immune evasion through immune checkpoint signaling. In addition, SASP can regulate epithelial–mesenchymal transition (EMT) and cancer stem cell (CSC)-associated traits via multiple signaling axes, including IL-6/STAT3, TGF-β/SMAD, and Wnt/β-catenin, leading to the enhancement of tumor invasiveness, metastatic potential, and therapeutic resistance. However, these effects are highly context-dependent, with their specific outcomes determined by the composition of SASP, its duration, and the state of the tumor microenvironment [99,100,101]. The dual role of SASP in therapy-induced senescent cells is shown in Figure 2.

## 8. Regulation of Tumor Cell Fate Decisions: Coordinated Actions of Intrinsic and Extrinsic Factors

### 8.1. Intrinsic Factors: Coupled Regulation by Genetic, Epigenetic, and Metabolic Programs

From the perspective of intrinsic genetic determinants, the p53/p21 and p16/RB pathways represent central tumor-suppressive axes governing cell cycle arrest, senescence, apoptosis, and tumor escape. DNA damage and cellular stress signals activate the p53 pathway, leading to the induction of p21 expression and subsequent inhibition of cell cycle progression. In parallel, the p16/RB pathway plays a critical role in the establishment and maintenance of the senescent state, reinforcing the stability of cell cycle arrest [53]. When these key genes are mutated or functionally inactivated, cells are more prone to bypass growth suppression, thus shifting toward tumor-promoting fates. For instance, studies in Ercc1-deficient mouse models have shown that loss of p53 disrupts p21-mediated cell cycle regulation, exacerbating liver pathology and polyploidization [102].

Epigenetic states provide an additional layer of plasticity in cell fate determination. Alterations in chromatin architecture and DNA methylation landscapes regulate the expression of genes involved in cell cycle control, inflammatory responses, and stress signaling. These processes involve diverse histone modifications, such as H3K9me3 and H3K27me3, which modulate chromatin compaction and accessibility, thereby influencing transcription factor binding and contributing to intratumoral heterogeneity [103,104].

Cellular metabolism, as a fundamental determinant of cell function, also plays a crucial role in cell fate regulation [105]. Enhanced glycolysis in tumor cells can alter the NAD^+^/NADH ratio and the levels of key metabolic intermediates, thereby influencing epigenetic modifications [106]. Recent studies have further demonstrated that metabolic reprogramming can directly regulate chromatin accessibility and cell fate through NAD^+^-dependent histone deacetylation [107]. Meanwhile, mitochondrial dysfunction and the accumulation of reactive oxygen species (ROS) can induce DNA damage responses and activate stress-related signaling pathways such as p38 MAPK, which, under certain conditions, promote the entry of cells into the senescence program [108].

The NAD^+^/sirtuin pathway is a critical hub linking cellular metabolism, epigenetic regulation, and cellular aging. The sirtuins (SIRT 1–7) constitute a family of NAD^+^-dependent protein-modifying enzymes with activities in lysine deacetylation, ADP-ribosylation, and/or deacylation. These enzymes participate in cellular stress responses and regulate gene expression, DNA damage repair, metabolic homeostasis, and cell survival [109]. During aging, alterations in NAD^+^ levels can affect sirtuin activity, thus further reshaping the epigenetic landscape and senescence-associated transcriptional programs [19]. Studies have shown that the SIRT7-mediated deacetylation of H3K18 is closely associated with the maintenance of oncogenic transformation [110]. In cancer, the roles of sirtuins are highly context-dependent and dualistic. SIRT1, SIRT2, and SIRT3 can exert either tumor-promoting or tumor-suppressive functions in different tumor contexts, with SIRT3 more commonly acting as a mitochondrial tumor suppressor. SIRT6 predominantly functions as a tumor suppressor; however, under specific therapeutic pressures or in particular tumor settings, it may also support cancer cell survival. SIRT7 is generally considered pro-tumorigenic in most studies. Therefore, therapeutic targeting of the NAD^+^/sirtuin pathway should be evaluated in relation to the specific sirtuin family member, tumor type, disease stage, and treatment context [106,111].

### 8.2. Extrinsic Factors: Immune System, Tumor Microenvironment, and Therapeutic Pressure

The immune system represents a critical determinant of cell fate decisions. Under effective immune surveillance, senescent cells are recognized and eliminated by natural killer (NK) cells and CD8^+^ T cells through the expression of stress-induced ligands and the secretion of chemokines, thereby exerting antitumor effects. In contrast, under immunosuppressive conditions, SASP can promote the accumulation of regulatory T cells (Tregs) and myeloid-derived suppressor cells (MDSCs) via IL-6/STAT3 and TGF-β signaling, thereby inhibiting immune-mediated clearance and instead supporting tumor progression [112,113].

The tumor microenvironment (TME) also plays a pivotal role in shaping cell fate decisions. Tumor-associated fibroblasts, endothelial cells, and other stromal components influence tumor cell behavior through the secretion of a wide array of factors, with one key mechanism being the activation of intracellular signaling pathways that promote tumor cell growth and survival via paracrine signaling [114,115]. In addition, hypoxic conditions regulate gene expression through hypoxia-inducible factor-1α (HIF-1α), facilitating metabolic adaptation and enhancing invasive potential, while alterations in the extracellular matrix (ECM) modulate cell migration through mechanotransduction signaling [116].

Anticancer therapies, as exogenous selective pressures, exert dual effects on tumor cell fate. Chemotherapy and radiotherapy induce DNA damage responses that trigger therapy-induced senescence (TIS), leading to cell cycle arrest and the suppression of tumor growth. However, residual senescent cells can promote inflammatory responses, maintain cancer stem cell (CSC) properties, and induce immunosuppression through SASP secretion, thereby driving tumor recurrence and the expansion of resistant clones. Furthermore, therapeutic selection pressure may enrich for subpopulations of tumor cells with stem-like characteristics or enhanced immune evasion capabilities [13,117,118].

## 9. Therapeutic Strategies Targeting Senescent Cells in Cancer Treatment

Highly proliferative tumor cells are particularly susceptible to pro-senescence therapies, which suppress tumor growth by inducing stable cell cycle arrest. However, in certain contexts, persistently surviving senescent tumor cells can remodel the tumor microenvironment through SASP, thereby promoting the development of therapeutic resistance. If senescent cells are not effectively eliminated following treatment, a subset of these cells may escape the senescence program and re-enter the cell cycle, and may even acquire cancer stem cell-like phenotypes, ultimately serving as a critical source of tumor recurrence and drug resistance. Therefore, achieving precise control over the balance between the induction of senescence and the clearance of senescent cells has emerged as a key regulatory determinant of therapeutic outcomes in cancer treatment [37,119,120].

### 9.1. Common Therapeutic Modalities in Cancer Treatment

Common chemotherapeutic agents that induce cellular senescence include doxorubicin, etoposide, temozolomide, and docetaxel. Among these, etoposide induces senescence by targeting topoisomerase II, thereby impairing DNA synthesis and replication. Temozolomide, an alkylating agent, induces DNA crosslinking and triggers DNA damage-associated senescence [121,122,123]. Compared with chemotherapy, radiotherapy primarily relies on ionizing radiation and offers greater spatial selectivity, enabling the precise delivery of radiation to tumor sites while minimizing damage to surrounding normal tissues. Consequently, it has been widely applied across multiple tumor types [124]. Antibody–drug conjugates (ADCs) represent a targeted cancer therapy strategy in which monoclonal antibodies are chemically linked to cytotoxic agents. This approach facilitates the selective delivery of cytotoxic payloads to tumor cells, thus inducing senescence and apoptosis [125,126].

### 9.2. Senolytics

Senolytics are a class of pharmacological agents that selectively eliminate senescent cells [127]. The first generation of senolytic drugs—including Dasatinib (D), Quercetin (Q), Fisetin (F), and Navitoclax—were identified through hypothesis-driven research strategies. Dasatinib, a tyrosine kinase inhibitor approved for clinical use in the United States since 2006, has been shown to induce apoptosis in senescent cells and exhibits a favorable safety profile. Quercetin and fisetin are naturally occurring flavonoids found in fruits and other dietary sources. Navitoclax (ABT-263) is a small-molecule inhibitor targeting anti-apoptotic members of the BCL-2 family, including BCL-2, BCL-XL, and BCL-W. By disrupting the dependence of senescent cells on anti-apoptotic signaling, navitoclax induces mitochondrial pathway-mediated apoptosis, including in senescent tumor cells [128,129,130,131]. Advances in high-throughput library screening and related approaches have facilitated the development of next-generation senolytics. These strategies include the design of prodrugs and nanodelivery systems based on lysosomal activity and senescence-associated β-galactosidase (SA-β-gal), inhibition of the senescence-associated secretory phenotype (SASP), induction of apoptosis via Na^+^/K^+^-ATPase-dependent mechanisms, and immune-mediated clearance approaches such as chimeric antigen receptor (CAR) T cells, antibody–drug conjugates (ADCs), and vaccine-based strategies [132,133].

### 9.3. Senescence-Based Combination Antitumor Strategies

Cancer therapeutic strategies are gradually shifting from approaches that rely solely on senescence induction toward combinatorial strategies. Senescence-based combination therapies aim to first induce tumor cell senescence using pro-senescence treatments, followed by senolytics [12].

In this context, combining chemotherapeutic agents with senolytics has emerged as a representative therapeutic strategy. Conventional chemotherapeutic drugs, such as doxorubicin and cisplatin, induce therapy-induced senescence (TIS) in tumor cells, which can subsequently be targeted and eliminated by senolytics. This sequential approach, often referred to as the “one–two punch” strategy, has been proposed and validated in multiple preclinical models [37,120]. For example, Fereshteh Ahmadinejad et al., using head and neck squamous cell carcinoma models (HN30/HN12), demonstrated that cisplatin treatment induces senescence in tumor cells in vivo [134]. Subsequent administration of the senolytic agent navitoclax promoted the apoptosis of senescent tumor cells, resulting in delayed tumor growth and prolonged survival in mouse models. However, the target specificity and in vivo safety profile of senolytics remain incompletely defined, and their clinical applicability and long-term benefits require further investigation. For instance, BCL-2 family inhibitors such as navitoclax are still limited by toxicity concerns in clinical settings [135,136].

**Figure 3:**
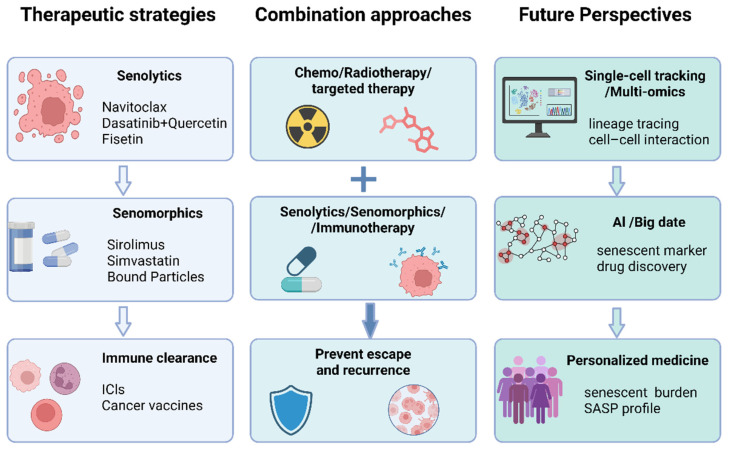
Therapeutic strategies and future directions. Strategies for targeting senescent cells involve senolytics, senomorphics, and immune-based approaches. Combination therapies aim to prevent tumor escape and recurrence. And future directions focus on single-cell analyses, AI-driven identification, and personalized medicine.

Another research direction centers on the integration of immunotherapy with senescence-targeting strategies. Senomorphics are a class of compounds developed to delay cellular senescence. They act primarily by suppressing SASP, which helps reduce the harmful effects of accumulated senescent cells. The effects of SASP are context-dependent, varying with both the type of senescence and the cellular environment. Consequently, interventions aimed at modulating or reprogramming SASP may provide valuable strategies for cancer therapy [137]. NF-κB is one of the central transcriptional regulators of the SASP. Activated NF-κB promotes the expression of multiple inflammatory SASP factors. From a therapeutic perspective, the NF-κB pathway can be regarded as a potential senomorphic target. Studies have shown that metformin inhibits the SASP by interfering with IKK–NF-κB activation, thereby reducing the release of inflammatory cytokines such as IL-6, IL-8, and CXCL5. And this may provide a new therapeutic target for the prevention and treatment of related diseases [138]. SASP exerts dual immunomodulatory effects, promoting immune cell recruitment and antigen presentation while also driving immunosuppression through the induction of immune checkpoint molecules such as PD-L1. Accordingly, integrating immune checkpoint blockade with SASP-targeting strategies may enhance the immune-mediated clearance of senescent and residual tumor cells [37,139,140,141,142]. In addition, innate immune components, including natural killer (NK) cells and macrophages, play critical roles in the immune surveillance of senescent cells. This suggests that reshaping or enhancing innate immune function to promote the clearance of senescent cells may represent a promising therapeutic avenue. However, these effects remain highly context-dependent across different tumor microenvironments [50,143]. Recent studies have shown that therapy-induced tumor cell senescence can establish an immunosuppressive barrier through the upregulation of PD-L1, whereas blockade of the PD-1/PD-L1 axis can reduce the burden of senescent cancer cells and suppress tumor recurrence by activating cytotoxic T lymphocytes [80]. Furthermore, Manuel Colucci et al., in preclinical mouse models of prostate cancer (PCa), demonstrated that docetaxel alone induces an SASP with tumor-promoting suppressive features [144]. In contrast, the combination of the retinoic acid receptor (RAR) agonist adapalene with docetaxel reprograms SASP, enhancing antitumor cytokines such as IL-12, IL-15, and IL-33 while suppressing metastasis-associated factors. This combinatorial approach also improves NK cell-mediated tumor clearance and further enhances tumor suppression by eliminating senescent tumor cells. Figure 3 shows cancer therapeutic strategies and future directions.

## 10. Conclusions and Future Directions

Cellular senescence has transitioned from a traditionally defined irreversible, quiescent endpoint to a highly dynamic process regulated by systemic factors, particularly the microenvironment. From the perspective of tumor initiation and progression, senescent cells can function as a tumor-suppressive barrier by inhibiting aberrant cell proliferation. Conversely, they may also promote tumor progression by secreting the senescence-associated secretory phenotype (SASP), which remodels the tumor microenvironment and drives inflammatory responses, immune evasion, and tumor development. Therefore, classifying cellular senescence simply as either tumor-suppressive or tumor-promoting is inherently limited. A more accurate view is that its biological functions are highly context-dependent, exhibiting pronounced spatiotemporal variability and tissue specificity, and should be considered a complex regulatory process with dual effects across different physiological and pathological conditions [51].

Notably, current research on cellular senescence largely relies on population-averaged analyses or a limited set of markers, which is insufficient to fully capture the heterogeneity of distinct senescent cell subpopulations during the senescence process, as well as their dynamic associations with tumor initiation, progression, and therapeutic responses [145]. The integration of single-cell multi-omics technologies, such as scRNA-seq, scATAC-seq, and spatial transcriptomics, will facilitate the dissection of the distribution patterns, lineage relationships, cell–cell interactions, and functional states of senescent cell subpopulations within the tumor microenvironment [146,147]. In addition, longitudinal studies integrating intravital imaging and lineage tracing approaches can facilitate the temporal dissection of state transitions and fate trajectories of senescent cells and their progeny, including dynamic processes such as immune clearance, phenotypic remodeling, senescence escape, and pro-tumorigenic evolution. However, analyses of senescence escape and therapeutic interventions have primarily focused on cancer cell populations, with a lack of data for tracking and analyzing individual cells. Future research may further advance toward single-cell characterization and dynamic tracking [148,149,150].

At the level of clinical translation, senescence-associated features provide a novel entry point for personalized cancer therapy. The proportion and functional states of senescent cells vary significantly across patients, age groups, and tumor types. Notably, in elderly populations, the higher burden of senescent cells contributes to a more complex influence on the tumor immune microenvironment, treatment tolerance, and the risk of recurrence. Therefore, future therapeutic strategies should incorporate patient-specific contexts and advance precision medicine approaches stratified by senescence-related biological characteristics. For instance, combining senolytics or senomorphics with immunotherapy, chemotherapy, or radiotherapy may exert synergistic effects in selected patient subgroups, thus improving overall treatment response rates and clinical outcomes [120,151,152].

Furthermore, the integration of artificial intelligence (AI) technologies is expected to serve as a key driver for advancing this field. By integrating large-scale clinical datasets, multi-omics data, and imaging information, AI holds the potential to develop models for the identification and functional prediction of senescent cells. Through the incorporation of multidimensional features, such as molecular biomarkers, the proportion of senescent cells, and their functional states, AI can enable quantitative and precise assessment of senescence levels within tissues, particularly facilitating dynamic evaluation of senescence states in the tumor microenvironment. In parallel, machine learning-based large-scale models may be applied to predict individual patient responses to different senescence-targeting therapeutic strategies, thereby informing personalized treatment decisions. Moreover, AI-assisted drug discovery platforms may accelerate the development of novel agents targeting senescence-related pathways [153,154,155,156].

Overall, future directions in the study of cellular senescence and cancer are likely to be characterized by a paradigm shift from static to dynamic perspectives, from population-level analyses to single-cell resolution, from mechanistic exploration to clinical translation, and from experience-based approaches to data-driven frameworks. Through interdisciplinary integration, particularly with the incorporation of artificial intelligence and precision medicine, these advances are expected to enable a more systematic understanding of the relationship between senescence and tumor biology, ultimately providing more forward-looking and individualized strategies for cancer prevention and therapy [157].

## Data Availability

No new data were created or analyzed in this study.
